# Comparison of multiple rebond shear strengths of debonded brackets after preparation with sandblasting and CO_2_ laser

**DOI:** 10.15171/joddd.2016.024

**Published:** 2016-08-17

**Authors:** Mojgan Kachoei, Amir Mohammadi, Maziar Esmaili Moghaddam, Sahand Rikhtegaran, Mahmoud Pourghaznein, Sajjad Shirazi

**Affiliations:** ^1^Associate Professor, Department of Orthodontics, Faculty of Dentistry, Tabriz University of Medical Sciences, Tabriz, Iran; ^2^Assistant Professor, Department of Orthodontics, Faculty of Dentistry, Tabriz University of Medical Sciences, Tabriz, Iran; ^3^Assistant Professor, Department of Orthodontics, Faculty of Dentistry, Urmia University of Medical Sciences, Urmia, Iran; ^4^Assistant Professor, Department of Restorative Dentistry, Faculty of Dentistry, Tabriz University of Medical Sciences, Tabriz, Iran; ^5^Assistant Professor, Department of Orthodontics, Faculty of Dentistry, Qom University of Medical Sciences, Qom, Iran; ^6^Research Fellow and Lecturer, Dental and Periodontal Research Center, Tabriz University of Medical Sciences, Tabriz, Iran; ^7^Biotechnology Research Center, Tabriz University of Medical Sciences, Tabriz, Iran

**Keywords:** Bond strength, CO_2_ laser, rebonding, sandblasting

## Abstract

***Background.*** Failure of orthodontic bracket bonds is a common occurrence during orthodontic treatment. Different techniques have been suggested in the literature to remove resin residues from the bracket bases and enamel surfaces to prepare the surfaces again after debonding. This study attempted to compare multiple rebond shear strengths (SBS) of debonded brackets following preparation with sandblasting and CO_2_ laser.

***Methods.*** The brackets were bonded on 30 human and bovine maxillary central incisors using self-curing composite resin. SBS was measured using Hounsfield testing machine. The brackets were rebonded for two other times after composite resin residues on their surfaces were removed, either with air abrasion or CO_2_ laser. The debonded brackets and enamel surfaces were also evaluated after each debonding procedure under a stereomicroscope in order to determine adhesive remnant index (ARI). SBS of debonded brackets after each step were compared between sandblast and CO_2_ laser groups.

***Results.*** We observed significant differences in SBS values between pre-recycling and first (P = 0.04), second (P = 0.007) and third recycling (P = 0.007) with laser. Recycling with sandblasting resulted in a decrease in SBS after the first and second recycling procedure; however, the SBS increased after the third recycling procedure, with no significant differences.

***Conclusion.*** SBS of brackets after recycling with sandblasting and laser beams was not significantly different, and both were at a favorable level. However, repeating the recycling procedure with sandblasting resulted in more favorable SBS compared to laser.

## Introduction

Fixed orthodontic treatment depends on an effective bond between brackets and enamel surfaces. Failure of orthodontic bracket bonds is a common occurrence during orthodontic treatment, with reports varying between 3.5% and 23%.^1,2^Debonding of brackets during treatment is an unpleasant occurrence for the clinician and the patient^3^ and results in an increase in treatment costs and duration.^4^ Various techniques have been suggested in the literature for removing resin residues from the bracket base or enamel surface and prepare the surfaces again after debonding, including the use of scalers or bond-removing pliers, different kinds of tungsten carbide burs, sandblasting and a variety of lasers.^1,2,5-15^, In this context, studies have been carried out on some techniques, including micro-etching, sandblasting and burning, with the aim of removing residual composite resin from the base of debonded brackets in order to use the brackets again.^16^ A study by Sonis did not show statistically significant differences in shear bond strengths (SBS) of a control group consisting of new brackets and the test group consisting of metallic brackets debonded from the enamel surface, which underwent air abrasion and were rebonded to the enamel surface.^17^ This resulted in the acceptance of this technique as a standard for rebonding of metallic brackets.^18^

Although the bond strength in brackets rebonded with sandblasting is comparable to the initial bond strength, previous studies have noted that sandblasting results in changes at bracket base.^1^ In addition, problems have been reported with this technique, including the need for facial masks and eye protection devices.^13^

Lasers used to remove resin residues include Er:YAG, Nd:YAG, XeCL Excimer, Er,Cr:YSGG and CO_2_.^5,11,14,19-21^, Electron microscope studies have shown that elimination of resin by laser and its depth can be controlled contrary to other techniques^22^and because of sufficient bond strength of brackets rebonded after laser preparation, this technique might be an acceptable alternative for sandblasting technique or use of new brackets, without infliction of extra costs.

Alexander et al^11^ showed that it is possible to selectively eliminate resin residues with certain wavelengths of Nd:YAG laser. Therefore, if the bond strength of brackets prepared with laser is sufficient and the bracket base does not undergo any changes, use of the laser technique is preferable to sandblasting. Therefore, evaluation of the laser technique as the most important innovation of this in vitro study will lead to another promising clinical application of lasers in dentistry if satisfactory results are achieved. It was hypothesized that use of new teeth for each bonding procedure and use of only one composite resin system in the present study would result in more precise results. Few studies to date have evaluated the SBS of orthodontic brackets after re-preparation of brackets and removal of resin residues by lasers. In addition, literature review showed that SBS of orthodontic brackets has not been evaluated for the third and fourth times. SBS values at third and fourth times were evaluated in the present study.

This study was carried out to evaluate the SBS of brackets after re-preparation of brackets and removal of resin residues, either with CO_2_ laser or sandblasting, and to study SBS values at third and fourth times.

## Methods

The study protocol was approved by the ethics committee of Tabriz University of Medical Sciences (Ref. No. 1389-295).

Employing previous data with a desired power of 90% at a significance level of 5%,^23-25^ a sample size of 11 pairs was needed to detect a difference of 2.53 between the two means, which was increased to 15 pairs in order to allow for probable losses.

This in vitro study was conducted on 30 standard Edge Wise brackets with grooved base (Ortho-Organizer, Carlsbad, California, USA) for maxillary central incisors, bonded to bovine teeth, in the Department of Orthodontics, Faculty of Dentistry, Tabriz University of Medical Sciences.

The teeth were stored in 0.1% aqueous thymol solution until used for the purpose of this study.^26^ The inclusion criteria for the teeth were absence of fractures or cracks visible by naked eye or under illumination and presence of a flat labial enamel surface. Teeth without these criteria were excluded from the study. The inclusion criteria were separately evaluated by two of the authors.^26^

The teeth were placed in self-curing acrylic resin molds, with their entire buccal surfaces out of the acrylic resin and parallel with the mold floor.^1^ Subsequently, the enamel surfaces were cleaned with fluoride-free pumice and a nylon brush in a low-speed handpiece for 5 seconds, followed by 10 seconds of rinsing with water.

The middle portions of the crowns of all the samples were etched with 37% phosphoric acid (3M Unitek, Manrovia, Califonia, USA) for 60 seconds and rinsed for 30 seconds with water. Then the samples were dried gently with air from a distance of 2 cm for 10 seconds so that the white chalky view appeared on the enamel.^1^ A thin layer of Unite Bonding System autopolymerizing bonding agent (3M Unitek, USA) was applied on the etched enamel surface. In addition, a thin layer of this bonding agent was applied on the base of the bracket of the maxillary central incisor (Orth-Organizer); then, a small amount of no-mix self-curing composite resin (Unite Bonding System, 3M Unitek, USA) was placed on the base of the bracket and the bracket was placed at the middle of the tooth surface. Point pressure was applied to the middle of the bracket, using a dental explorer and extra composite resin was removed using an explorer.^1^

The samples were stored in distilled water at 37ºC in an incubator for one week until the SBS tests were carried out.^1,27,28^ Then the samples were tested in a universal testing machine (Hounsfield Universal Teat Equipment, Model H5KS, Surrey, UK). The acrylic mold was placed on the jig of the machine.^1,27^ A stainless steel piston attached to the equipment, with a cutting edge and a cross-section of 0.5 mm, was placed at the bracket‒tooth interface parallel to the surface of tooth and the bracket base. The bracket was debonded by the application of a shearing force by the piston at a strain rate of 0.5 mm/min. Maximum force at failure was recorded and the SBS value was calculated using the following formula:^29^

Shear bond strength value (MPa) = Force (N) / Bracket surface area (mm^2^)

Subsequently, using the software program of the website www.randomization.com the debonded brackets were randomly divided into two groups with 15 teeth, based on surface preparation and composite resin residue removal technique.

**Group 1 (control):** Composite resin residues on the bracket surface were removed by air abrasion technique (Dento-Prep Ronving, Denmark) with 50-μ aluminum oxide particles from an approximate distance of 5 mm from the surface with visual observation of composite resin removal.^1,2^ Subsequently, rebonding procedure was carried out using new and un-bonded teeth using the same bonding technique as the initial one.^1^

**Group 2:** Composite resin residues on the bracket surface were removed with CO_2_ laser at a wavelength of 10600 nm and a 3-W output power, for 15 seconds and with a 1-mm distance from the surface. Then rebonding procedure was carried out using the same technique as the initial bonding procedure.

After rebonding of the samples, they were stored in distilled water at 37ºC in an incubator for one week until the test procedures.^1,27,28^Debonding was carried out in each group using a universal testing machine (Dento-Prep Ronving, Denmark) and bond strength data for each sample was recorded.^1,27^ All the bonding, debonding and bond strength measurement procedures were carried out by one operator to avoid bias. The operator was blinded to bracket preparation protocols.

Then all the samples in each subgroup were bonded for the third and fourth times using the protocol of that group and each bonding procedure was carried out on new intact teeth. SBS data was once again recorded for the third and fourth bonding procedures. Bond strengths for the first and second procedures were analyzed statistically.

The debonded brackets and enamel surfaces were evaluated after each debonding procedure at ×10 under a stereomicroscope (Coolpix Optical Stereomicroscope, Nikon, Japan) in order to determine adhesive remnant index (ARI)^19,27^ and distributions of various ARIs were reported.

The amount of remaining resin on tooth surfaces was described using ARI. The ARI scores ranged from 0 to 3 as follows: 0, no adhesive remaining on the tooth; 1, less than half of the enamel bonding site was covered with adhesive; 2, more than half of the enamel bonding site was covered with adhesive; 3, the enamel bonding site was covered entirely with adhesive.^19^

Evaluation of normal distribution of data was carried out by Shapiro-Wilk analysis, and repeated-measures ANOVA and post hoc Bonferroni test were used to evaluate the effect of recycling with sandblasting and laser on SBS. Spearman’s analysis was used to investigate the correlation between ARI and SBS. All the analyses were conducted with SPSS (Ver. 13) and P < 0.05 was considered significant.

## Results

Table 1 presents the mean SBS values in the sandblast and laser groups. Shapiro-Wilk analysis showed normal distribution of data in all the subgroups. In addition, Mauchly’s test of sphericity showed the absence of sphericity assumption.

**Table 1:** Means and standard deviations of shear bond strength values after three recycling procedures with sandblast technique and laser

**Group**	**Time of recycling**	**Mean** ±** SD**	**95% Confidence interval** **(Lower‒Upper)**
**Sandblast**	Pre-recycling	32.45 ± 2.01	28.13**‒**36.77
	First	25.58 ± 3.22	18.67**‒**32.49
	Second	26.34 ± 2.19	21.62**‒**31.05
	Third	30.75 ± 1.96	26.53**‒**34.96
**Laser**	Pre-recycling	35.42 ± 2.52	30.01**‒**40.83
	First	24.86 ± 1.98	20.60**‒**29.11
	Second	22.76 ± 1.78	18.94**‒**26.59
	Third	22.76 ± 1.51	19.51**‒**26.02

**Figure 1.**
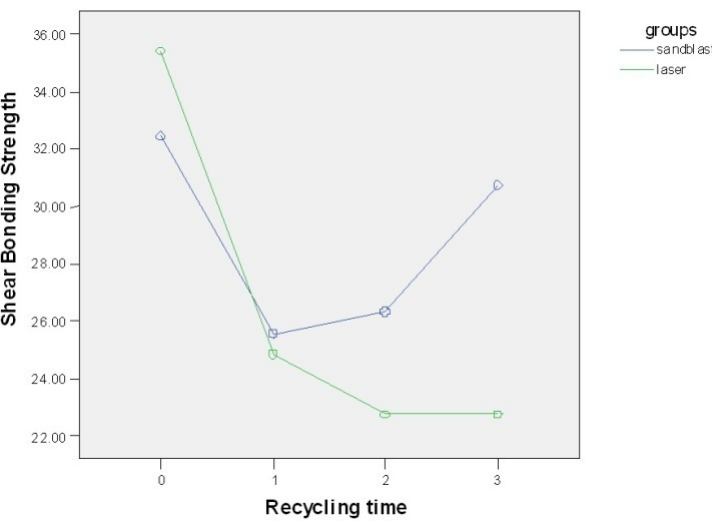
Shear bond strength values in the sandblast and laser group.

SBS gradually decreased in the laser group at different time intervals (Figure 1). Repeated-measures ANOVA with Greenhouse-Geisser correction revealed that the SBS values were significantly different between the time intervals after recycling with laser (F=10.90, P<0.0005).

Recycling with sandblasting resulted in a decrease in bond strength after the first recycling procedure; however, the bond strength increased after the second and third recycling procedures (Table 1; Figure 1). The changes in SBS during recycling with sandblasting were not statistically significant (Table 2).

**Table 2:** Post hoc Bonferroni pair-wise comparison of shear bond strength in the sandblast and laser groups

**Group**	**Comparison of different times of rebonding**	**Mean difference** ± **Std. error**	**P-value**
**Sandblast**	pre vs. first	6.86 ± 3.40	0.37
	pre vs. second	6.11 ± 2.43	0.14
	pre vs. third	1.70 ± 2.39	1.00
	first vs. second	−.75 ± 3.27	1.00
	first vs. third	−5.16 ± 3.04	0.66
	second vs. third	-4.41 ± 1.64	0.10
**Laser**	pre vs. first	10.56 ± 3.36	0.04^*^
	pre vs. second	12.65 ± 3.09	0.007^*^
	pre vs. third	12.65 ± 3.12	0.007^*^
	first vs. second	2.09 ± 2.59	1.00
	first vs. third	2.09 ± 2.09	1.00
	second vs. third	001 ± 1.37	1.00

Statistically significant (P < 0.05)

In addition, repeated-measures ANOVA showed that the mean SBS was not significantly different between the laser and sandblast groups with repeated recycling (F=1.32, P=0.26).

A post hoc test with Bonferroni correction revealed that recycling with laser resulted in a decrease in SBS from the pre-recycling phase to the first recycling (Table 1) (P=0.043). However, the decrease in SBS during the second and third recycling with laser was not statistically significant (Table 2).

The results of evaluation of debonded brackets and enamel surfaces after each debonding in relation to ARI are shown in Table 3. There was no significant correlation between SBS and ARI within each study group. However, an indirect and weak correlation was detected between total SBS and ARI scores (r_s_ = −0.27, P = 0.003).

**Table 3:** Frequencies of different adhesive remnant index (ARI) values in the sandblast and laser groups at different stages

**ARI**	**Sandblast recycling**				**Laser recycling**			
	**Before recycling**	**1st**	**2nd**	**3rd**	**Before recycling**	**1st**	**2nd**	**3rd**
0	8	4	4	1	9	5	4	0
1	3	6	4	1	5	4	0	4
2	4	4	4	6	1	0	0	4
3	0	1	3	7	0	6	11	7

## Discussion

Failure of orthodontic bracket bonds occurs frequently during orthodontic treatment.^1,2^ For economic reasons, brackets that are debonded during the orthodontic treatment are generally rebonded after removal of composite adhesive by means of different methods like sandblasting, mechanical grinding, adhesive burning and lasers.^30^ Some researchers have reported higher initial bond strength values compared to secondary bracket bond strength values^13,31,32^ and some others have reported higher bond strength values for secondary bonding procedures.^1,2,8,26,33^

Discrepancies in the results of different studies have been attributed to different reasons, including re-use of debonded teeth or new teeth, the technique used to remove residual resin from the debonded brackets or tooth surfaces, differences in bonding system and the composite resin, and not separating these confounding variables.^1,26^ In previous studies, overlapping results from these systems and absence of proper separation between them by statistical tests have resulted in errors in measurements. The aim of this study was to evaluate the effectiveness of CO_2_ laser in removing the adhesive from debonded bracket bases and to compare it with sandblasting. Three rounds of debonding procedure and use of new teeth in each step were considered to reduce bias in this study.

In the present study, the mean SBS values in the sandblast and laser groups were 28.7 and 26.4 MPa, which are higher than the scores reported in other studies for SBS before or after recycling. This might be attributed to the use of new teeth in each bonding procedure and use of only one composite resin type and also use of bovine teeth instead of human teeth in the present study compared to other studies. In the sandblast group, mean SBS for new brackets was more than that of recycled brackets and SBS increased after 2 and 3 times of recycling. However, the differences in SBS between groups with pre-recycling and groups with 1, 2 and 3 times of recycling were not significant. In addition, an increase in bond strength in the third recycling in the sandblast group might be due to an increase in the porosity of the base of the brackets caused by sandblasting used in the present study which is in line with the results of other studies.^1,23^ In laser groups, a decreasing pattern in SBS from new brackets to 3 times of recycling was noted. Differences in SBS in pre-recycling (new brackets) group and other laser subgroups were significant.

The results of the present study were different from those of Yassaei et al,^34^ in which the SBS of composite resin decreased significantly after application of CO_2_ laser to remove composite resin. This discrepancy might be attributed to differences in bracket and composite resin types and possibly to differences in bracket alloys.

Ishida et al,^35^ and Chacko et al^36^ showed the efficacy of this laser in removing the composite resin remaining on the bracket base and achieving favorable bond strength during re-bonding of brackets. The results of the present study in relation to the effect of CO_2_ laser on decreasing the SBS during the second recycling of brackets were consistent with the results of Ishida et al.^35^

The quantity of remaining adhesive after bracket debonding is clinically important. More stresses will occur at the enamel surface when the amount of remaining adhesive on the tooth surface is reduced, or when bond failure occurs at the area closer to enamel and adhesive region.^37^ On the other hand, adhesive remaining on bracket base decreases the contact area between meshwork and adhesive, resulting in a decrease in SBS value.^34^ However, no significant correlation was detected between SBS and ARI in the present study. Besides, ARI indices increased from pre-recycling to third recycling in both groups, which can be attributed to the increased interlock between the bracket base and adhesive owing to the used techniques.^34^

The present study did not show any significant differences in bracket bond strength values after recycling with sandblasting and laser beams, with both at a favorable level. Our findings were consistent with those of Yassaci et al,^34^who evaluated the efficacy of Er,Cr:YSGG laser and sandblasting in eliminating composite resin from bracket bases. However, use of CO_2_ laser compared to the use of sandblasting resulted in a decrease in SBS, which was contrary to the results reported by Yassaci et al.^34^

## Conclusion

There were no significant differences in bracket bond strength values after recycling with sandblasting and laser beams, with both at a favorable level. However, repeating the recycling with sandblasting resulted in more favorable SBS compared to laser.

## Acknowledgments

The authors would like to thank the staff at the Dental Biomaterial Research Center at Tabriz University of Medical Sciences for their assistance in carrying out the study.

## Authors’ contributions

MK and SS contributed to the concept and design, and critically revised the manuscript. ME, SR and MP contributed to data acquisition and interpretation, and drafted the manuscript. AM consulted on and performed statistical evaluation. AM, MK and SS contributed substantially to discussion. All the authors have read and approved the final manuscript.

## Funding

This study was supported and funded by Tabriz University of Medical Sciences.

## Competing interests

The authors declare no competing interests with regards to the authorship and/or publication of this article.

## Ethics approval

The study protocol was approved by the ethics committee of Tabriz University of Medical Sciences (Ref. No. 1389-295).

## References

[1] Mui B, Rossouw PE, Kulkarni GV (1999). Optimization of a procedure for rebonding dislodged orthodontic brackets. Angle Orthod.

[2] Eminkahyagil N, Arman A, Cetinsahin A, Karabulut E (2006). Effect of resin removal methods on enamel and shear bond strength of rebonded brackets. Angle Orthod.

[3] Hobson RS, Hogg SD (2001). Bond strength to surface enamel for different tooth types. Dent Mater.

[4] Powers JM, Turner DS (1997). Orthodontic adhesives and bond strength testing. Semin Orthod.

[5] Brian W, Thomas C (1996). Thomas CLaser- aided degradation of composite resin. Angle Orthod.

[6] Gwinett A, Gorelick L (1977). Microscopic evaluation of enamel after debonding. Am J Orthod Dentofac Orthop.

[7] Hong YH, Lew KK (1995). Quantitative and qualitative assessment of enamel surface following five composite removal methods after bracket debonding. Eur J Orthod.

[8] Pickett KL, Sadowsky PL, Jacobson A, Lacefield W (2001). Orthodontic in vivo bond strength: comparison with in vitro results. Angle Orthod.

[9] Campbell PM (1995). Enamel surfaces after orthodontic bracket debonding. Angle Orthod.

[10] Pus MD, Way DC (1980). Enamel Loss due to orthodontic bonding with filled and unfilled resins using various clean- up techniques. Am J Orthod.

[11] Alexander R, Xie J, Friod D (2002). Selective removal of residual composite from dental enamel surfaces using third harmonic of a Q-switched Nd: YAG laser. Lasers Surg Med.

[12] Rouleau BD Jr, Marshall GW Jr, Cooley RO (1982). Enamel surface evaluations after clinical treatment and removal of orthodontic brackets. Am J Orthod Dentofac Orthop.

[13] Canay S, Kocadereli I, Akca E (2002). The effect of enamel air ablation on the retention of bonded metallic orthodontic brackets. Am J Orthod Dentofac Orthop.

[14] Dumore T, Fried D (2000). Selective ablation of orthodontic composite by using sub microsecond IR laser pulses with optical feedback. Lasers Surg Med.

[15] Zachrisson BU, Artun J (1979). Enamel Surface appearance after various debonding techniques. Am J Orthod Dentofac Orthop.

[16] Mui B, Rossouw PE, Kulkarni GV (1999). Optimization of a procedure for rebonding dislodged orthodontic brackets. Angle Orthod.

[17] Sonis AL (1996). Air abrasion of failed bonded metal brackets: a study of shear bond strength and surface characteristics as determined by scanning electron microscopy. Am J Orthod Dentofacial Orthop.

[18] Zachrisson BU, Buyukyilmaz T (2005). In: Graber TM, Vanarsdall RL, Vig KW, eds. Orthodontics: Current Principles and Techniques. 4Th edition ed.

[19] Basaran G, Ozer T, Berk N, Hamamci O (2007). Etching enamel for orthodontics with an Erbium, Chromium:Yttrium-Scandium-Gallium-garnet laser systme. Angle Orthod.

[20] Yow L, Nelson JS, Berns MW (1989). Ablation of bone and PMMA by an XeCl 308 nm excimer laser. Lasers Surg Med.

[21] Yassaei S, Fekrazad R, Shahraki N, Goldani Moghadam M (2014). A comparison of shear bond strengths of metal and ceramic brackets using conventional acid etching technique and Er:YAG laser etching. J Dent Res Dent Clin Dent Prospects.

[22] Fuhrmann R, Gutknecht N, Magunski A, Lampert F, Diedrich P (2001). Conditioning of enamel with Nd: YAG and CO2 dental laser systems and with phosphoric acid. J Orofac Orthop.

[23] Montero MM, Vicente A, Alfonso-Hernández N, Jiménez-López M, Bravo-González LA (2015). Comparison of shear bond strength of brackets recycled using micro sandblasting and industrial methods. Angle Orthod.

[24] Shirazi S, Kachoei M, Shahvaghar Asl N, Shirazi S, Sharghi R. Arch width changes in patients with Class II division 1 malocclusion treated with maxillary first premolar extraction and non-extraction method. J Clin Exp Dent. 10.4317/jced.52840 PMC5045687 27703608

[25] Mirzakouchaki B, Shirazi S, Sharghi R, Shirazi S (2016). Assessment of Factors Affecting adolescent Patients’ Compliance with Hawley and Vacuum Formed Retainers. J Clin Diagn Res.

[26] Montasser M, James L, Drummond C, Evans A (2008). Rebonding of orthodontic brackets part i, a laboratory and clinical study. Angle Orthod.

[27] Bishara SE, Oonsombat C, Soliman MM, Warren JJ, Laffoon
 JE
, Benjamin J (2005). Comparison of bonding time and shear bond strength between a conventional and a new integrated bonding system. Angle Orthod.

[28] Bishara SE, Laffoon JF, Warren JJ (2000). The effect of repeated bonding on the shear bond strength of a composite resin orthodontic adhesive. Angle Orthod.

[29] Ahmadizenouz G, Esmaeili B, Taghvaei A, Jamali Z, Jafari T, Amiri Daneshvar F, Khafri S (2016). Effect of different surface treatments on the shear bond strength of nanofilled composite repairs. J Dent Res Dent Clin Dent Prospects.

[30] Wendl B, Muchitsch P, Pichelmayer M, Droschl H, Kern W (2011). Comparative bond strength of new and reconditioned brackets and assessment of residual adhesive by light and electron microscopy. Eur J Orthod.

[31] Regan D, LeMasney B, van Noort R (1993). The tensile bond strength of new and rebonded stainless steel orthodontic brackets. Eur J Orthod.

[32] Wright WL, Powers JM (1985). In vitro tensile bond strength of reconditioned brackets. Am J Orthod Dentofac Orthop.

[33] Retief DH, Denys FR (1979). Finishing of enamel surfaces after debonding of orthodontic attachments. Angle Orthod.

[34] Yassaei S, Aghili H, KhanPayeh E, Goldani Moghadam M (2014). Comparison of shear bond strength of rebonded brackets with four methods of adhesive removal. Lasers Med Sci.

[35] Ishida K, Endo T, Shinkai K, Katoh Y (2011). Shear bond strength of rebonded brackets after removal of adhesives with Er,Cr:YSGG laser. Odontology.

[36] Chacko PK, Kodoth J, John J, Kumar K (2013). Recycling stainless steel orthodontic brackets with Er:YAG laser - An environmental scanning electron microscope and shear bond strength study. J Orthod Sci.

[37] Mirzakouchaki B, Shirazi S, Sharghi R, Shirazi S, Moghimi M, Shahrbaf S (2016). Shear bond strength and debonding characteristics of metal and ceramic brackets bonded with conventional acid-etch and self-etch primer systems: An in-vivo study. J Clin Exp Dent.

